# Vitamin D intoxication: case report

**DOI:** 10.1590/S1679-45082014RC2860

**Published:** 2014

**Authors:** Tatiana Aporta Marins, Tatiana de Fátima Gonçalves Galvão, Fernando Korkes, Domingos Augusto Cherino Malerbi, Arnaldo José Ganc, Davi Korn, Jairo Wagner, João Carlos de Campos Guerra, Wladimir Mendes Borges, Fábio Teixeira Ferracini, Hélio Korkes

**Affiliations:** 1 Hospital Israelita Albert Einstein, São Paulo, SP, Brazil.

**Keywords:** Vitamin D/poisoning, Hypercalcemia, Case reports

## Abstract

Hypervitaminosis D is a rarely reported condition. In general it is only perceived when hypercalcemia is not resolved. The use of vitamin D has increased in recent years because of its benefits, but as a result, intoxication cases have occurred more frequently. This report describes a patient who presented worsening of renal function and hypercalcemia. After investigation, vitamin D intoxication was confirmed and it was due to an error in compounding.

## RESUMO

A hipervitaminose D é pouco relatada. Geralmente, só é percebida quando o quadro de hipercalcemia não se resolve. Em razão de seus benefícios, o uso de vitamina D aumentou nos últimos anos; consequentemente, os casos de intoxicação também tiveram aumento. Este relato destacou um caso em que o paciente apresentava piora da função renal e hipercalcemia. Após investigação, ficou confirmada a intoxicação por vitamina D devido a um erro de manipulação da droga.

## INTRODUCTION

Clinical conditions associated to vitamin D intoxication have been scarcely reported since hypercalcemia suggests other presumptive diagnoses.^(1)^ The number of cases has recently increased because of more prescriptions of vitamin D to treat hypovitaminosis D. There are few of these products traded by the pharmaceutical industries, leading to increased demand for compounded medications, which may lead to compounding errors, such as the case herein reported. The patient developed hypercalcemia and worsening of renal failure due to ingestion of a dose 2,000-fold higher than what was prescribed.

## CASE REPORT

A 53-year old male patient, with history *diabetes mellitus*, hypertension, non-dialysis chronic renal failure and smoking. He was admitted to hospital in September 2011 to investigate worsening of the renal function, pruritus, muscle weakness, lack of appetite and weight lost. Table 1 displays the results of the biochemical tests before hospitalization.

**Table 1:** Biochemical tests before hospitalization

Month/year	Calcium (8.4-10.4) mg/dL	Ionized calcium (1.14-1.31) mmol/L	Parathyroid hormone (12.0-65.0) pg/mL	Creatinine (0.6-1.2) mg/dL
March/2011	9.0	–	–	1.30
July/2011	14.7	–	–	3.71
August/2011	–	2.11	18.0	3.84
September/2011	13.4	–	–	4.67

On the initial physical examination, the following were measured: weight 86kg, height 1.93cm, blood pressure 140x70mmHg and heart rate 80bpm. The biochemical test results were creatinine 67mg/dL, calcium 13.4mg/dL, ionized calcium 1.88mmol/L, parathyroid hormone (PTH) 15.3pg/mL. The ultrasound showed renal calculi, one was obstructing and the urinary tract was dilated. He was operated on for double-J catheter implanting and ureterolithotripsy. A 3-cm-long colon neoplasm was observed in colonoscopy. Parathyroid scintigraphy, myelogram and positron- emission tomography (PET) scan presented normal results.

The following management was prescribed: hyperhydration; administration of furosemide, corticosteroid and iron; and replacing erythropoietin by methoxy polyethylene glycol-epoetin beta due to suspected skin allergy. Intranasal and subcutaneous calcitonin was also introduced.

Hypercalcemia persisted even after exeresis of colorectal adenocarcinoma. Investigating parathyroid adenoma by ultrasound, scintigraphy and biopsy ruled out primary hyperparathyroidism. Biochemical test results were creatinine 2.69mg/dL, calcium 13.0mg/dL, ionized calcium 1.67mmol/L, PTH 17.5pg/mL, vitamin D >100ng/mL.

The laboratory tests for bone metabolism were inconclusive, and PTH inappropriately persisted with “normal” levels even with hypercalcemia. The laboratory diagnosis was probably influenced by renal failure or possible vitamin D intoxication, considering the patient took the vitamin for some months before admission to hospital, at 2,000IU per day. Since hypervitaminosis was suspected, renal function, as well as serum calcium and vitamin D levels were monitored, and ibandronate sodium was introduced and later replaced by cinacalcet.

The drug capsules were submitted to laboratory analysis due to suspected intoxication. The analytical methodology and specification used were based on the United States Pharmacopea (USP 34) Oil-soluble vitamins capsules, by means of high-performance liquid chromatography. The amount of vitamin D found was two-thousand-fold higher than expected for each capsule.

As shown in table 2, the laboratory results dropped and vitamin D levels decreased slowly as expected, because of its elimination profile.^(1,2)^

**Table 2:** Biochemical tests after discharge from hospital

Month/year	Calcium (8.4-10.4) mg/dL	Ionized calcium (1.14-1.31) mmol/L	Parathyroid hormone (12.0-65.0) pg/mL	Creatinine (0.6-1.2) mg/dL	25(OH)D (30-100) ng/mL
January/2012	12.0	1.63		2.48	226
February/2012	10.7	1.51		2.40	196
March/2012	10.0	1.37	25.2	3.50	70
June/2012		1.49		2.40	

25(OH)D: vitamin D.

## DISCUSSION

Vitamin D supplementation has currently been more often used for increasing calcium absorption in the body and reducing the risk of diseases, such as rickets and osteoporosis. Some recent studies have shown that vitamin D deficiency is associated to an increased risk of developing cardiovascular diseases, some types of cancer, in addition to inflammatory and autoimmune diseases.^(3–5)^ The body can produce vitamin D only after sunlight exposure. When regular sunlight exposure is not frequent, only foods are not enough to keep appropriate levels of the vitamin. Hence supplementation with medications is necessary. The recommended dose varies from 800 to 4,000IU per day according to age.^(5,6)^

Vitamin D supplements can be easily purchased over the counter, in the form of ergocalciferol or cholecalciferol, in diverse formulations and dosages. The cases of hypervitaminosis D usually occur in excessive supplementation.^(3,7,8)^ The upper limit of daily vitamin D intake needed to cause toxicity is unknown; however, up to 10,000IU per day was considered safe in a healthy population.^(3)^

The estimated toxic dose of vitamin D should be greater than 100,000IU per day for, at least, one month.^(9)^ In the present case, the dose used was 40-fold higher than that recommended. There are reports of clinical conditions in which the amount was 8- and 18-fold the maximum recommended dose.^(9)^ The initial tests showed increased calcium levels and undetectable PTH. Later the intoxication was confirmed since the serum vitamin D level was over 1,000ng/mL. In another report, a patient was diagnosed as acute renal failure secondary to hypercalcemia, and had normal PTH and increased vitamin D levels.^(3)^ Investigating the medications the patient was taking, it was observed that the vitamin D3 vial contained 50,000IU per capsule instead of 1,000IU. Moreover, to aggravate the situation, the patient took two more supplements containing vitamin D3; thus, he was taking approximately 50,400IU per day.

The diagnosis of vitamin D intoxication is not common in cases of hypercalcemia, and it was less often made before availability of the vitamin for supplementation. This condition is likely to be associated to primary hyperparathyroidism, multiple myeloma or other neoplasms. The patient reported was paradoxical because in case of hypercalcemia, PTH levels would tend to be low or, in typical hyperparathyroidism, they would be high. However, the patient's PTH levels were normal and hindered diagnosis; hence it was necessary to investigate all possible causes of hypercalcemia.

The patient took a compounded cholecalciferol supplement. Overdose was found by laboratory analysis of the capsules that contained 4,000,000IU instead of 2,000IU per capsule. This kind of compounding error may occur mainly when dealing with very-low dose products; in such cases, the pure input is not weighed, it is just diluted. Vitamin D is particularly produced at a high potency – 1mg is equal to 40,000IU. Therefore, the pharmacist must take care as required to perform correct dilution, weighing and conversion procedures. The compounding pharmacies should encourage good practices, establish quality criteria and strictly supervise all stages of the production process. The pharmacy seriously failed and was notified as a preventive measure to avoid new events

## CONCLUSION

Vitamin D supplementation should be appropriately monitored due to potential risk of intoxication, especially when made by compounding.
